# Social robotics for children: an investigation of manufacturers’ claims

**DOI:** 10.3389/frobt.2023.1080157

**Published:** 2023-12-19

**Authors:** Jill A. Dosso, Anna Riminchan, Julie M. Robillard

**Affiliations:** ^1^ Neuroscience, Engagement, and Smart Tech (NEST) Laboratory, Department of Medicine, Division of Neurology, The University of British Columbia, Vancouver, BC, Canada; ^2^ Neuroscience, Engagement, and Smart Tech (NEST) Laboratory, British Columbia Children’s and Women’s Hospital, Vancouver, BC, Canada

**Keywords:** social robot, health, child development, consumer information, internet, social interaction, parents, emotion

## Abstract

As the market for commercial children’s social robots grows, manufacturers’ claims around the functionality and outcomes of their products have the potential to impact consumer purchasing decisions. In this work, we qualitatively and quantitatively assess the content and scientific support for claims about social robots for children made on manufacturers’ websites. A sample of 21 robot websites was obtained using location-independent keyword searches on Google, Yahoo, and Bing from April to July 2021. All claims made on manufacturers’ websites about robot functionality and outcomes (*n* = 653 statements) were subjected to content analysis, and the quality of evidence for these claims was evaluated using a validated quality evaluation tool. Social robot manufacturers made clear claims about the impact of their products in the areas of interaction, education, emotion, and adaptivity. Claims tended to focus on the child rather than the parent or other users. Robots were primarily described in the context of interactive, educational, and emotional uses, rather than being for health, safety, or security. The quality of the information used to support these claims was highly variable and at times potentially misleading. Many websites used language implying that robots had interior thoughts and experiences; for example, that they would love the child. This study provides insight into the content and quality of parent-facing manufacturer claims regarding commercial social robots for children.

## 1 Introduction

Socially assistive robots can provide companionship, facilitate education, and assist with healthcare for diverse populations, including children. We define social robots as possessing three elements: sensors to detect information, a physical form with actuators to manipulate the environment, and an interface that can interact with humans on a social level (del Moral et al., 2009). Social robots’ interactions with humans have four key aspects; 1) they are physical, 2) they can flexibly react to novel events, 3) they are equipped to realize complex goals, and 4) they are capable of social interaction with humans in pursuit of their goals (Duffy et al., 1999).

Child-specific uses of social robots in healthcare include providing support during pediatric hospitalization (Okita, 2013; Farrier et al., 2020), reducing distress during medical procedures (Trost et al., 2019), mitigating the effects of a short-term stressor (Crossman et al., 2018), and acting as a social skills intervention for children with Autism Spectrum Disorder (Diehl et al., 2012; Pennisi et al., 2016; Prescott and Robillard, 2021). While research on social robots has increased over the last decade, results have oftentimes been inconclusive, mixed, or limited due to small sample sizes (Dawe et al., 2019; Trost et al., 2019). Existing work has also been limited by a restricted focus on highly developed countries, the study of a limited number of robotic platforms, implementation of a heterogenous set of control conditions and outcome measures, and a lack of transparency in reporting (Kabaci et al., 2021). Previous research on social robots has predominantly occurred in clinical (Ullrich et al., 2016) or laboratory contexts (Crossman et al., 2018). Some individual social robots have received extensive investigation from the scientific community, often with a particular focus on children with autism (Beran et al., 2015; Arent et al., 2019; Rossi et al., 2020). Due to rapid turnover in the commercial robot market, there are relatively few studies that focus on social robots currently available for purchase as researchers oftentimes modify existing commercial social robots to better suit their experimental goals (Ullrich et al., 2016; Tulli et al., 2019). Social robots’ intended uses in the real world have received minimal investigation, and a description of the larger environment of child-specific commercial social robot functionality and impact is lacking from the scientific literature. The present study aimed to characterize how robots are marketed towards child consumers.

There are potential ethical ramifications of building social robots for children. Some have argued that the use of social technologies may diminish human-human interaction (Turkle, 2011), that ascribing moral standing to social robots is problematic (Coeckelbergh, 2014), and that deception is integral to human-robot relationships (Sætra, 2020). Others have suggested there is little evidence that introducing social robots reduces human interaction and that social isolation is not due to robots but to systematic and societal issues in how social needs are valued (Prescott and Robillard, 2021). It has been proposed that social robots may instead act as a “social bridge” to friends, relatives, and teachers of children (Dawe et al., 2019; Prescott and Robillard, 2021). The question of whether robots are “overhyped” has also been considered (Parviainen and Coeckelbergh, 2021), as many highly anticipated social robotics start-ups have had difficulty transferring technological breakthroughs in research fields to commercial and industrial applications (Tulli et al., 2019).

Despite this debate, COVID-19 has accelerated the demand for social robots as people seek to maintain social interaction while reducing potential disease exposure (Scassellati and Vázquez, 2020; Tavakoli et al., 2020; Zeng et al., 2020; Prescott and Robillard, 2021). When deciding whether to invest considerable sums of money into a social robot for a child, parents and caregivers are likely to seek information about these devices through the internet. Studies of how robots are incorporated into everyday life identify different stages of social robot acceptance. The first of these is an expectation or pre-adoption stage (de Graaf et al., 2018; Sung et al., 2010). In this stage, potential buyers seek to gather more information about the technology, form an idea of its value to them, and finally begin to create expectations for the technology, often using the internet as a tool for information gathering. Therefore, the content of online information about child-specific robots is likely to form a large part of potential users’ expectations for these devices. Despite the growing market for social robots, there is little knowledge of what parents and caregivers are exposed to when making decisions around robot adoption. To address this knowledge gap, we: 1) described the current landscape of social robots available to children from a prospective consumer’s perspective, 2) captured and assess the claims made by manufacturers around their functionality, and 3) evaluated the quality of the evidence supporting these claims. Qualitative analysis of manufacturer claims, in the manner done here, and evidence quality evaluation using the QUEST tool have been successfully used to understand the markets for health-related topics including wearable brain technologies, e-cigarettes, infant formula, dementia prevention, and prescription drugs (Kaphingst et al., 2004; Perry et al., 2013; Yao et al., 2016; Robillard and Feng, 2017; Coates et al., 2019; Pomeranz et al., 2021).

## 2 Materials and methods

### 2.1 Sampling

Google, Yahoo, and Bing, the most popular search engines in North America, were searched using a combination of keywords for “child” and “social robot,” as well as their synonyms (“kid,” “teen,” “youth,” “pediatric,” “paediatric,” “adolescent,” “robotic,” “social robot”). This method allowed the researchers to take the perspective of a consumer looking for social robots marketed towards children via the internet. The IP address used was in Vancouver, British Columbia, localizing search results to North America, and the personalization of search results was minimized using strict privacy settings and blockers.

Based on search engine user behaviour, the first three pages of search results from each engine, excluding advertisements, were considered (Beitzel and Jensen, 2007). Each search result page was loaded and manually screened for the names of potential social robots.

**FIGURE 1:**
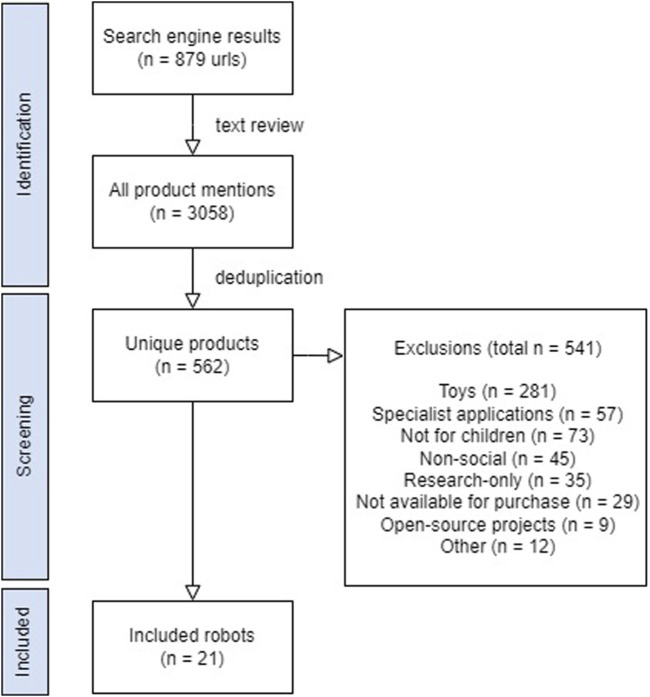
Identification process for eligible robots and their manufacturer websites.

Inclusion criteria for robots to be included in the study were: 1) product is consistent with above-stated definition of a social robot; 2) product is targeted towards consumers 18 years of age or younger; 3) product is physically embodied and ready-to-use; and 4) product is commercially available for purchase or pre-order in North America at the time of the search or was out-of-stock after previously being available. Exclusion criteria were that the robot: 1) was not directed towards children or did not have any child-specific functionality; 2) was sold on secondary marketplace websites (e.g., Amazon, Ebay); 3) required consumer-initiated communication with the manufacturer (e.g., a direct email) as opposed to a manufacturer-created step towards purchasing the robot (e.g., a price quote or inquiry website field for the consumer to fill out, a “buy robot” page); and 4) was considered one of the following robot types: telepresence robots, open-access 3D printable robots, research-only robots, prototypes, and crowdfunded robots. A full list of inclusion and exclusion criteria can be found in Supplementary Table S1. These inclusion criteria were developed iteratively by all authors based on an agreed-upon definition of “social robot”—sensors, actuators, and a social interface—and through discussion of sample products. The final sample was achieved through consensus from all authors (Figure 1). Only the initial reason to exclude a product was documented. Figure 1 reports each product only once, despite the fact that many products would have met multiple criteria for exclusion had they been screened further (e.g., surgical robots are also not targeted to children, nor are they available to the public to easily purchase).

### 2.2 Claim analysis

Websites were reviewed, and claims were extracted and coded, during the period of April 12–July 6, 2021. Manufacturer claims around the social robots marketed towards children were collected by examining the manufacturer’s websites in full, which were archived at the time of coding. The text of each website was then coded for sentences that made claims about the robot. The inclusion criteria were that the claim: 1) was related to the child specific uses or impacts of the robot; 2) was explicitly stated in the text 3) pertained to the social robot, the purchase-maker (typically the parent/guardian), or the child/children. The claim was not included if it only described the social robot’s physical features (e.g., degrees of freedom, touch sensors, cameras), as these were not informative in terms of the social robot’s claimed benefits or functionality. It was also not included if it was a customer testimonial as researchers did not consider this a direct manufacturer claim. For each claim, we identified the user being referenced (e.g., parent vs. child), the theme of the claim via content analysis, and the specific topic of the claim.

Claim themes were classified by first coding the specific topic around which each claim centered, often the verb of the manufacturer’s sentence (e.g., coding “communication” if manufacturer claim stated “robot will help your child communicate more effectively”). These specific topics were then grouped into several themes. In this example, communication was grouped into the “Interaction” theme, which also included specific manufacturer claim topics such as collaboration and listening.

The quality of each website as a whole was characterized using a subset of items from the Quality Evaluation Scoring Tool (QUEST) for online health information (Robillard et al., 2018). Data were analyzed and visualized in R using tidyverse packages (Wickham et al., 2019). The complete dataset is available at https://osf.io/nbj9t/.

## 3 Results

**FIGURE 2:**
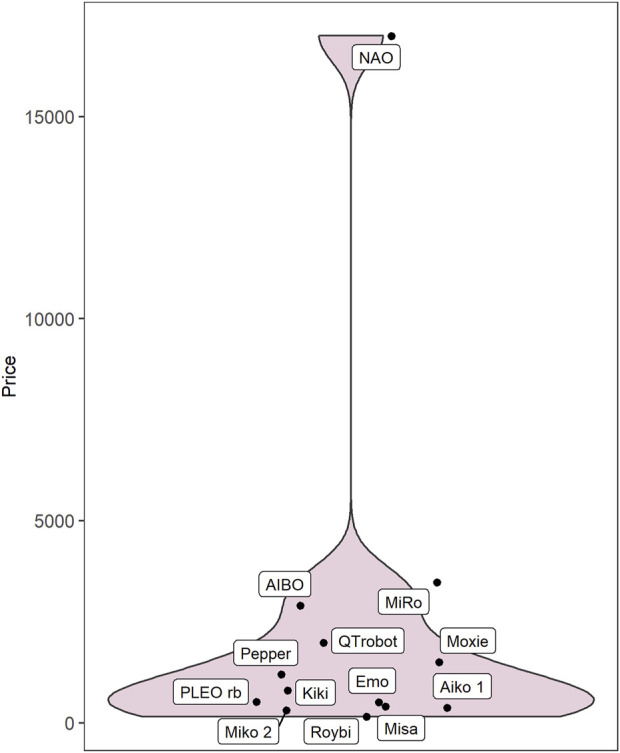
Retail prices listed for commercially available social robots for children.

On the whole, robots that met all criteria for inclusion were frequently mentioned in our web searches (occurring in 26.7 unique web results on average), while excluded products were mentioned less frequently (3.6 results). Twenty-one robots met our inclusion criteria. They were manufactured in the United States (*n* = 6), Japan (*n* = 4), China (*n* = 3), France (*n* = 2), Spain (*n* = 2), and India, Luxembourg, Taiwan, and the United Kingdom (*n* = 1 each). Where it was possible to identify robot prices from manufacturer’s websites (13 of 21 robots), they ranged from 150 to 17,000 USD, with a median price of 799 USD (Figure 2).

### 3.1 Manufacturer claims

**FIGURE 3:**
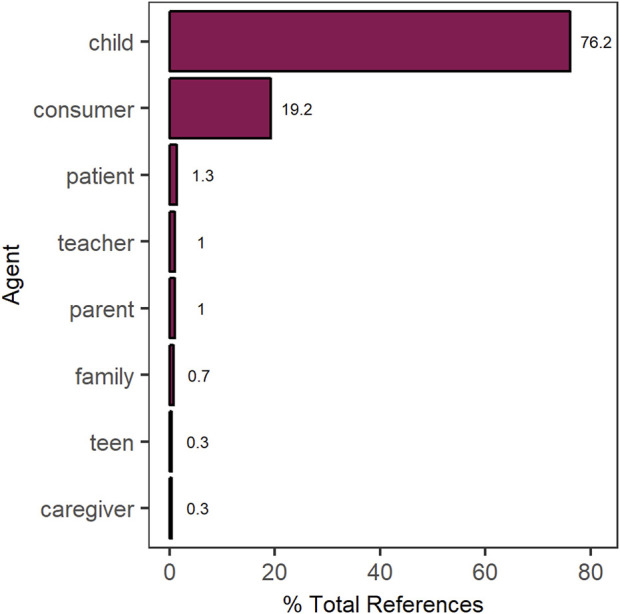
Distribution of references made to different individuals across the set of websites.

A total of 653 individual claims were identified, with a mean of 31 claims per robot (range: 5–75). For each claim, we identified the individual being referenced (Figure 3). Most claims focused on what the robot could do and how the child would benefit from social robot intervention. For example, “Aiko also could be your mentor to learn and develop your cognitive, emotional and social skills.” A minority of claims were centered around what the robot could do for a general consumer, e.g., “BUDDY is your personal assistant.” Claims referenced others, like parents or teachers, only rarely.

We used inductive content analysis to organize these claims into larger themes. The research team reviewed a random sample of claims (approximately 10% of the sample or 65 claims) as a group to develop an initial coding guide. This was applied to a new, similarly sized set of statements by one coder, who then brought their results and any edge cases to the group for discussion. This process was repeated several times until all authors felt that the coding guide was robust, at which point a single coder coded the entire sample.

**FIGURE 4:**
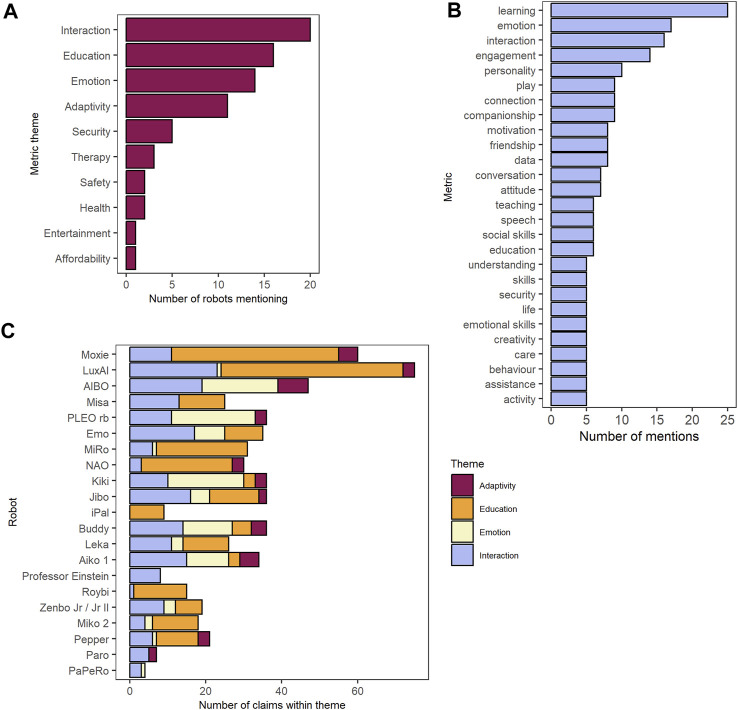
**(A)** Number of robots mentioning each metric theme at least once. **(B)** Metrics occurring for five or more claims. **(C)** Number of claims per robot for each of the four most common metric themes.

We identified eight major themes into which all claims fell (Figure 4A). These were Education (*n* = 251 claims), Interaction (*n* = 205), Emotion (*n* = 111), Adaptivity (*n* = 41), Health and Wellbeing (*n* = 20), Safety and Security (*n* = 19), Entertainment (*n* = 5), and Affordability (*n* = 1). Education claims centered on new or improved outcomes that could develop as a result of using the robot. For example, “QTrobot is an expressive social robot designed to increase the efficiency of special needs education … ” Interaction claims displayed the social robots’ capabilities of exchanging information with consumers or its environment. They also included the robot’s impact on the consumer’s interactions with the robot itself, and other humans, such as “[u]se Zenbo Lab to create interactive conversations and activities to help students practice speaking and listening skills.” Emotional claims predominantly illustrate the social robot’s capabilities to express emotion, or its effects on the emotions of the consumer: “[Kiki] is fully aware of her surroundings and expresses a diversity of emotions and reactions.” Finally, Adaptivity referred to claims revolving around the social robot’s ability to grow and learn from its interactions with the world, e.g., “Curiosity drives aibo, with new experiences fusing fun and learning together into growth. It’s these experiences that shape aibo’s unique personality and behavior.”

For each robot, we identified the theme for which the largest number of claims were made. Metrics were coded directly from the manufacturer’s own wording whenever possible, and were converted to singular nouns (e.g., “children have learned” would be coded as “learning”; “has many actions” would be coded as “action”). We collected 290 unique metrics (Figure 4C). Those which occurred more than four times in the total sample are shown in Figure 4B. The five most common were learning [“(t)his results in more attention and concentration from children and helps them to learn more effectively”], emotion (“BUDDY has a range of emotions that he will express naturally throughout the day based on his interactions with family members”), interaction (“BUDDY connects, protects and interacts with every member of your family”), engagement (“Leka makes it easier to keep each child engaged and motivated”), and personality (“Aibo even has likes and dislikes—another dimension of its personality”).

### 3.2 Evidence quality

**FIGURE 5:**
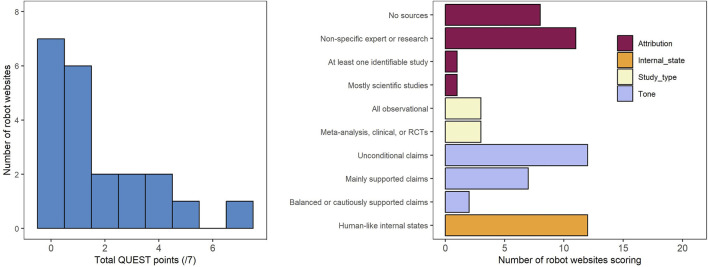
Quality Evaluation Scoring Tool (QUEST) scores for content of robot websites.

We evaluated the quality of the manufacturers’ claims (Figure 5) using a subset of relevant items from the QUEST instrument (Robillard et al., 2018). First, we looked at claim attribution–whether and how websites referenced sources. Eight websites mentioned no sources (receiving a score of 0). Eleven mentioned expert sources or research findings that could not be traced to a specific study, provided links to sites, advocacy bodies, or similar (score of 1). One referenced at least one identifiable scientific study (score of 2). One referenced mainly (>50% of claims) identifiable scientific studies (score of 3).

Next, we considered the types of studies that were mentioned by manufacturers. Three websites included mention of all observational work (score of 1), and three mentioned studies that were meta-analyses, randomized clinical trials, or clinical studies (score of 2). We also considered the tone of the claims that were made. Twelve websites used language that supported their claims without conditions (score of 0). Language included terms like “will,” “guarantee,” “does,” and there was no discussion of limitations. Seven websites made mainly supported claims, using language like “can reduce” or “may improve,” but with no discussion of limitations (score of 1). Two websites made claims with balanced or cautious support (score of 2), with statements of limitations or mention of findings that contradict claims of efficacy.

Finally, while not part of the QUEST instrument, we also coded websites for the presence of language attributing human-like internal states like emotions or motivations to the robot. We found these types of statements on 12 of 21 websites. Examples included: “A robot that *understands how you feel* and cheers you on…,” “*He will be happy* to give you a warm welcome to come home to … ,” [the robot] “*loves making friends* and is $\left[\ldots \right]$ a *naturally inquisitive* robot,” “*Loves you back*” [all italics by the authors].

## 4 Discussion

Our analysis showed that the marketing of current commercial social robots was focused on their potential to act as emotional companions or educators for children. However, the quality of the information used to support these claims was highly variable and at times potentially misleading, such that parent-facing information about social robots for children may not be an accurate representation of the strength of the evidence in this area.

There is some evidence for the efficacy of social robots on each of the four main application dimensions identified in our sample (adaptivity, education, emotion, and interaction). However, the quality of the claims we documented is not aligned with the available evidence. Nineteen of the twenty-one websites we evaluated had no statements of limitations on their claims at all, and these claims were sometimes broad (e.g., “This smart robot will perform brilliantly with many services useful to all”). The gap between robot capabilities and user expectations likely contributes to the current limited success of commercial social robots (Tulli et al., 2019). Below, we consider the four largest themes we identified and existing research evidence for each.

The most common theme among claim statements was interactivity; statements were that the robot was interactive, capable of play, companionship, connection, and friendship. The child-robot interaction research literature does indeed prioritize questions of interactivity and social behaviour (Belpaeme et al., 2013; Di Dio et al., 2020; Parsonage, 2020), and the responsivity of a social robot has been shown to change human behaviour and willingness to use the devices (Birnbaum et al., 2016). However, the claims we documented (e.g., “a predictable and tireless new friend,” “she’s your loyal companion,” “develops a familiarity with people over time”) suggest long-term, intimate relationship formation between children and robots that is not evidence-based. For instance, a 2020 review of interactive social robotics work reports that most studies on robots for companionship take place over short time scales and focus on the best social cues for a robot to produce (Lambert et al., 2020). Relatively few studies look at the effect of human-robot interaction on the human user. Creating a truly interactive social robot is computationally very difficult, particularly if speech or emotion modeling are desired (Dosso et al., 2022). To circumvent these challenges, many studies of human-robot interaction rely on a Wizard-of-Oz methodology in which the device is controlled by a human operator. A 2021 scoping review of social robots as mental health interventions for children found only five papers featuring autonomously behaving robots, only two of which were speech-capable (Kabacińska et al., 2021). Market-ready devices vary in their degree of social immersiveness, but manufacturers claim that their robots can serve as a true friend to users run ahead of available data.

We also documented a number of claims based on robots’ putative educational functions, with language around supporting learning, providing motivation, teaching specific social and emotional skills, supporting creativity, and leading to academic success. Education has long been targeted as a potential application area for social robotics. A 2018 review of 101 articles on the subject reported positive outcomes of social robot use on cognitive (e.g., speed, number of attempts) and affective (e.g., persistence, anxiety) outcomes and documented different conceptualizations of a robot’s role in education: as tutor or teacher, peer, and novice (Belpaeme et al., 2018). However, they also point to logistical challenges of robot implementation, risks associated with delegating education to robots rather than human teachers, and technical limitations for existing robots. Educational robots were more effective when they targeted specific skills or topics, rather than being general purpose or used by multiple students. Relatedly, a 2020 systematic review found that robots were effective for supporting student engagement more so than teaching complex material (Lambert et al., 2020). Compared to the theme of interactivity, claims within the theme of education are perhaps better supported by the existing research literature. However, the particular robots being sold, and the fact that they are being marketed for unstructured home use, limits the generalizability of the data in the present study.

Turning next to the theme of emotion, we recorded a large number of claims that robots could feel love and happiness, have moods and feelings, were attuned to a user’s emotions, could cheer up the user or make them happy, and could be trusted. In terms of emotional impacts of social robots for children, there is evidence for mitigation of negative emotional responses and memories in children receiving vaccines, and robots may help with patient anxiety and pain perception (Beran et al., 2015; Trost et al., 2019). There has also been extensive research and protocols developed for modeling emotional systems in social robotics that mimic ones displayed by humans and animals (Paiva, 2014; Pessoa, 2017). However, research on social robots as mental health supports for adults has been recently described as “nascent,” with few conditions being studied and limited generalizability (Scoglio et al., 2019; Guemghar et al., 2022), and this is also true for children’s robots (Kabacińska et al., 2021). A 2022 review of work on robotic emotions identifies this as a topic of accelerating research interest (Stock-Homburg, 2022), but reports that studies are largely short-term, lab-based, and focused on robot development rather than a user’s own emotional responses to the encounter (e.g., asking people to rate a robot’s facial expression). As with the first two themes, we argue that manufacturer claims around robot emotion are overstated relative to available evidence. Interestingly, we noticed that many of the Emotion claims were about the robot’s own supposed inner experience (e.g., “loves you”). This may shed light on the intention of these websites–to create a fantasy narrative about how to relate to the product (i.e., to conceive of it as sentient and engaged in a relationship with the user) rather than making causal and factual claims about function.

The final major theme among the claims we examined was adaptivity. We noted statements that robots had evolving personalities, adaptable behaviour, and could learn, grow, and be customized. Models for social robot creation have incorporated behavioural adaptation -- the ability of the robot to grow and learn from social interactions (Salter et al., 2008). However, most existing work on this topic centers around robots adapting to a user’s performance on a pre-specified task, usually during a one-off interaction (Ahmad et al., 2017). Limited work on robot adaptation to simple elements of user personality, such as introversion, has been conducted (e.g., Tapus et al., 2008), and no studies on robots adapting to a user’s culture of demographic background were reported in a recent systematic review (Ahmad et al., 2017). As with the other themes, we argue that manufacturer claims about robots adapting to their child users, especially with the implication that robots detect complex features of the user like their temperament and personal quirks, are misaligned with the capabilities of existing robots.

There was often a mismatch between the strength of evidence for a particular robot and the degree to which their website referenced data and research. For example, one manufacturer’s website featured a page highlighting research on social robots titled “The Science Behind [Robot]”. The page contained graphics outlining results from peer-reviewed social robot research (conducted with robots other than the one being sold). The tone used in these claims meets the criteria for a rating of “0” for Tone on the QUEST scale, indicating full support of the claims by mostly using non-conditional verb tenses (“can,” “will”), with no discussion of limitations. While the website did not claim that these studies’ results pertained to their robot specifically, they used graphics in the robot’s signature colour scheme and intermixed the text with videos, pictures, and the silhouette of the robot. By contrast, the website of one of the most-researched social robots (NAO) made very few references to research or evidence. These two examples together illustrate the difficult task that faces consumers if they would like to evaluate the strength of evidence for social robots based on the information available from manufacturers. In this work, we only examined whether social robot claims referenced published work; we did not evaluate the quality of that published work itself, or the presence of sources of bias in this published work. An examination of the types of evidence manufacturers reference is a worthy area of investigation for future study. Furthermore, our analysis did not evaluate published work for sources of bias (e.g., funded or conducted by robot manufacturers versus independent investigators).

This analysis reveals the priorities of robot manufacturers, which is helpful for the Human-Robot Interaction (HRI) research community. First, the claim attribution analysis shows that HRI work is not being put in front of consumers in these contexts very often, pointing to an opportunity for improved science communication. Second, we see that social robots are offered primarily around the 800 USD price point, suggesting that researchers who want to study robots that may plausibly enter the home should focus their attentions on this band of devices. Third, we note that health and safety are mentioned by relatively few websites, in contrast with the focus on these themes in the scientific literature. This may mean that devices supporting these functionalities tend not to be marketed to families (instead to medical institutions, for example), or that these technologies are not yet market-ready. Finally, it is interesting to observe that emotion was a top-three metric theme for manufacturers’ claims. Computational models of human-robot emotional alignment are still emerging (e.g., Dosso et al., 2022) and this work suggests that manufacturers see this as a priority.

Recommendations for adults looking to purchase robots for children are lacking. Online resources about purchasing smart toys remind consumers to vet toys’ privacy capabilities before purchasing, know toy features, be wary of hacking and data misuse, look out for cheap toys with poor safety features, educate children about digital privacy, focus on reputable companies and retailers, and look at certifications like COPPA or the Federal Trade Commission’s kidSAFE Seal (InternetMatters, 2023; Miranda, 2019; Fowler, 2018; Best, 2022; Gummer, 2023; Gallacher and Magid, 2021; Safe Search Kids, 2022; Knorr, 2020).

We propose three additional recommendations based on our analysis and the unique properties of social robots as a child-facing product. We encourage potential customers to: 1) pay attention to the tone of the claims made in promoting a product; qualified language like “may support” and statements of limitations are indications of quality; 2) be cautious with claims that robots will adapt to the user, will respond to their emotions, or will create a long-term bond, as these statements are unlikely to be true on technical grounds, especially for robots at a low price point; 3) be aware that social robots for children is a product category with high turnover. Devices frequently go off the market and ongoing technical support once purchased is not guaranteed. Products with longer histories are more likely to be supported by more stable companies, but all devices should be purchased with caution.

A limitation of the current study is our focus on simulating the buyer’s experience. By using a search engine-based collection of robot names, we intended to mimic a potential consumer’s initial search process. While this process may not have captured every possible social robot in the “long tail” of the market, we feel confident that we identified the large majority of commercial social robots for children. Most robots included in our sample arose repeatedly in our search process. We chose to focus our study specifically on claims made by manufacturers themselves, rather than secondary sellers, to avoid cases where resellers might misunderstand the products, intentionally mislead consumers (e.g., due to sponsorships or paid partnerships), or over- or under-state robot capabilities. We also limited the analysis to robots that were currently available for purchase or easy to pre-order, excluding manufacturers’ websites listing defunct robots as simply “out of stock” or inviting visitors to “contact us.” These decisions were intended to create a sample of robots that were currently available and manufacturers’ current conceptions of their features and applications.

The present work establishes a baseline understanding of which social robots are currently being marketed for children and how manufacturers describe these devices and prompts several new lines of inquiry. One would be to gather more information on how consumers, particularly parents of children, make decisions around purchasing social robots. Another would be to consider in more detail the 290 metrics that were identified, many of which pointed to specific robot features. Which of these features are most important to parents and children when actually using the robot? Which are primary drivers of purchasing behaviour? Another topic arising in previous research is claims around the data security, privacy, and recording features of social robots (Chatterjee et al., 2021). Such claims were included in our sample and could be further investigated. Whether these claims are supported by the design of the robots, and the ethical implications of these design decisions, are outstanding questions.

Our results highlight the challenges faced by potential consumers when attempting to purchase a social robot for a child online. The information available from manufacturers is of mixed quality but shows that current social robots tend to be marketed as interactive, educational, emotional, and adaptive. Researchers have a responsibility to communicate high quality scientific information about the current state of social robotics to the public.

Manufacturer claims about child-facing social robots are not well grounded in evidence. This is likely to make consumers’ decision-making difficult. Furthermore, the research evidence that is available may not reflect the way that commercial devices are actually marketed and used. More research on the home-based applications of social robots, as well as greater transparency from manufacturers at the decision-making stage, are needed for consumers to be fully informed on the research-backed effects and uses of child-marketed social robots.

## Data Availability

The datasets presented in this study can be found in online repositories. The names of the repository/repositories and accession number(s) can be found in the article/Supplementary Material.
