# Supplementary material for: Social robotics for children: an investigation of manufacturers’ claims
Source: Front Robot AI. 2023 Dec 19;10:1080157. doi: 10.3389/frobt.2023.1080157 (PMC10770258; doi:10.3389/frobt.2023.1080157)
Supplement: Supplementary file 1 [file Table1.docx]

## Supplementary material for Dosso, Riminchan, & Robillard, 2023, “Social robotics for children: An investigation of manufacturers’ claims”

## Supplementary Table 1: Inclusion and Exclusion Criteria for Manufacturer Websites in Sample.

| **Inclusion Criteria** | **Exclusion Criteria** |
| --- | --- |
| Functionality targeted towards children 18 years or under. | Virtual-only, app-only, telepresence, research-only, prototype-only, cleaning, movement rehabilitation, or open-source robots. Voice assistants such as Alexa, Google Home, Siri, etc. |
| Product complies with definition of a social robot (Morgal, Parfo, & Angulo, 2009; Duffy, Rooney, & O’Hare, 1999) | Robots out of commission or otherwise permanently not available for sale. |
| Robot has a manufacturer website through which it is available for purchase (includes Kickstarter). | Robots available through Ebay, Amazon, or other reseller websites, without an independent manufacturer’s website. |
| Robot is commercially available in North America for sale, pre-sale, re-stock, or direct consumer inquiry through website. | Robot not directly sold to consumers, or not possible to obtain through direct communication with seller (e.g., via means such as direct email communication). |
